# Development of a Modified Three-Day T-maze Protocol for Evaluating Learning and Memory Capacity of Adult Zebrafish

**DOI:** 10.3390/ijms21041464

**Published:** 2020-02-21

**Authors:** Bui Thi Ngoc Hieu, Nguyen Thi Ngoc Anh, Gilbert Audira, Stevhen Juniardi, Rhenz Alfred D. Liman, Oliver B. Villaflores, Yu-Heng Lai, Jung-Ren Chen, Sung-Tzu Liang, Jong-Chin Huang, Chung-Der Hsiao

**Affiliations:** 1 Department of Chemistry, Chung Yuan Christian University, Chung-Li 32023, Taiwan; hieubtn90@gmail.com (B.T.N.H.); gilbertaudira@yahoo.com (G.A.); 2 Department of Bioscience Technology, Chung Yuan Christian University, Chung-Li 32023, Taiwan; nguyen021194@gmail.com (N.T.N.A.); stvn.jun@gmail.com (S.J.); stliang3@gmail.com (S.-T.L.); 3 Faculty of Applied Sciences, Ton Duc Thang University, Ho Chi Minh City 758307, Vietnam; 4 The Graduate School, University of Santo Tomas, Manila 1015, Philippines; rhenzalfredliman@gmail.com; 5 Department of Biochemistry, Faculty of Pharmacy and Research Center for Natural and Applied Sciences, University of Santo Tomas, Manila 1015, Philippines; obvillaflores@ust.edu.ph; 6 Department of Chemistry, Chinese Culture University, Taipei 11114, Taiwan; lyh21@ulive.pccu.edu.tw; 7 Department of Biological Science & Technology College of Medicine, I-Shou University, Kaohsiung 82445, Taiwan; jrchen@isu.edu.tw; 8 Department of Applied Chemistry, National Pingtung University, Pingtung 90003, Taiwan; 9 Center for Nanotechnology, Chung Yuan Christian University, Chung-Li 32023, Taiwan

**Keywords:** passive avoidance, ZnCl_2_, spatial memory, T-maze, leptin a, zebrafish

## Abstract

A T-maze test is an experimental approach that is used in congenital research. However, the food reward-based protocol for the T-maze test in fish has low efficiency and a long training period. The aim of this study is to facilitate the T-maze conditions by using a combination of the principles of passive avoidance and a spatial memory test. In our modified T-maze settings, electric shock punishment (1–2 V, 0.3–0.5 mA) is given at the left arm, with a green cue at the right arm. Also, the depth of both arms of the T-maze was increased. The parameters measured in our T-maze design were latency, freezing time, and time spent in different areas of the T-maze. We validated the utility of our modified T-maze protocol by showing the consistent finding of memory impairment in ZnCl_2_−treated fish, which has been previously detected with the passive avoidance test. In addition, we also tested the spatial memory performance of *leptin a* (*lepa*) mutants which displayed an obesity phenotype. The results showed that although the learning and memory performance for *lepa* KO fish were similar to control fish, they displayed a higher freezing behavior during the training phase. In conclusion, we have established a modified T-maze protocol that can be used to evaluate the anxiety, learning, and memory capacity of adult zebrafish within three days, for the first time.

## 1. Introduction

Rodents are the most popular animal models for neuroscience studies to represent complex neurological conditions such as depression, anxiety, and dementia with a simplified approach conducted in laboratory conditions [1,2,3]. Several behavioral assessment tools such as T-maze, Morris water maze, open field, and shuttle box have been developed for spatial memory, locomotion, and anxiety tests [4,5]. However, due to the 3Rs (Replacement, Reduction, and Refinement) and animal welfare requirements on reducing the sacrifice of mammalian animals, more alternative lower vertebrate and invertebrate models have been introduced to the behavioral research community in the recent years. For example, the use of fruit fly and fish for the investigation of brain mechanisms in behavioral neuroscience research has surged in recent years [6,7,8]. Locomotion, visual discrimination, food searching, social and shoaling behaviors of fish have received much attention due to their reportorial nature of behaviors of vertebrate animals [9,10,11]. As one of the most known animal models, zebrafish (*Danio rerio*) have many advantages such as high productivity, body transparency, and rapid embryo developmental processes that make them a practical and useful model organism for genetic and biomedical research. Over the past decades, zebrafish have been used extensively in studies ranging from developmental biology, toxicology, pharmacology, and behavioral experiments [12,13,14].

T-maze is an instrument that has been utilized to evaluate spatial learning and memory in rodents [15,16,17] and fish [11,18]. T-maze has also been applied to evaluate the effect of chemical pollution [19,20] or the pharmacological effect of drugs in the behavior of animal models [21,22,23]. In zebrafish, the T-maze test is based on food reward or stimulus with the conspecifics [18] (summarized in Table S1). However, the long training period, which usually lasts from eight to ten days, becomes one of the challenges to perform spatial learning and memory test in zebrafish. Thus, modifications in the T-maze method is necessary to increase its effectiveness and efficiency.

The specific aim of this study is to develop a rapid, direct, and reliable protocol by combining T-maze and passive avoidance test principles to evaluate learning and memory performance in adult zebrafish. Initially, we modified the T-maze setting and protocol with the aid of electric shock conditioning. Next, we tested the influence of gender and toxicity (due to Zn^2+^ overload) on zebrafish learning and memory performance in T-maze. Finally, the learning and memory performances of a genetic mutant zebrafish with a *leptin a* (*lepa)* gene deficiency were also evaluated in detail.

## 2. Results

### 2.1. Overview of the Modified T-Maze to Perform Conditioned Preference Test

**Figure 1:**
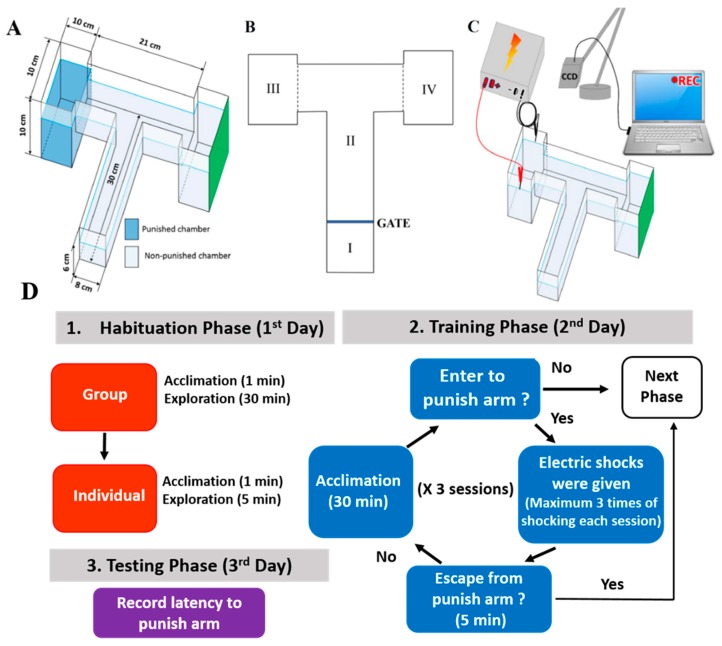
Experimental design of the T-maze apparatus in the zebrafish model. (**A**) Illustration of the T-maze dimensions. (**B**) Three sections were defined: the start chamber (Zone I), the novel arm (Zone II), and the choice arm (swim to Zones III and IV). The depth of water in the novel arm was maintained at 35 and 85 mm in two of the deeper choice arms (**C**) The T-maze was equipped with an electric shock device (1–2 V, 0.3–0.5 mA), video recorder, and the data were analyzed by GraphPad Prism. (**D**) Flow diagram of the T-maze protocol.

The design and instrumental setting of our modified T-maze are presented in Figure 1A–C. Our T-maze is based on the instrument described by Braida et al., [24] with some modifications. The T-Maze apparatus used in this study was made from transparent Plexiglas, including two deeper arms and one straight long arm (Figure 1A). This apparatus consists of Zone I (for habituation), Zone II (start chamber and novel arm) and Zones III and IV (deep water chambers) (Figure 1B). A green cue was given at Zone IV (right arm) to produce an unfavored stimulus that forces zebrafish to swim to the left arm during the habituation and training phases. When the fish moved into Zone III (left arm), a mild electric shock (1–2 V, 0.3–0.5 mA) was given as a punishment for spatial conditioning training.

The entire operation process, including the habituation (day 1), training (day 2), and testing (day 3) phases, are illustrated in Figure 1D. First, fish were acclimatized to the experimental conditions on day 1 to minimize apparatus novelty stress. Then, for the training trial on day 2, electric shocks were given when fish moved into the left arm. This punishment can force the fish to build up passive avoidance behavior (also known as spatial conditioning). Finally, in the testing phase, the memory retention of zebrafish was evaluated by measuring the latency to enter the left arm and time spent in the punishment arm by day 3 (Figure 1D).

### 2.2. The T-Maze Paradigm in Enhancing Place Conditioned Preference

**Figure 2:**
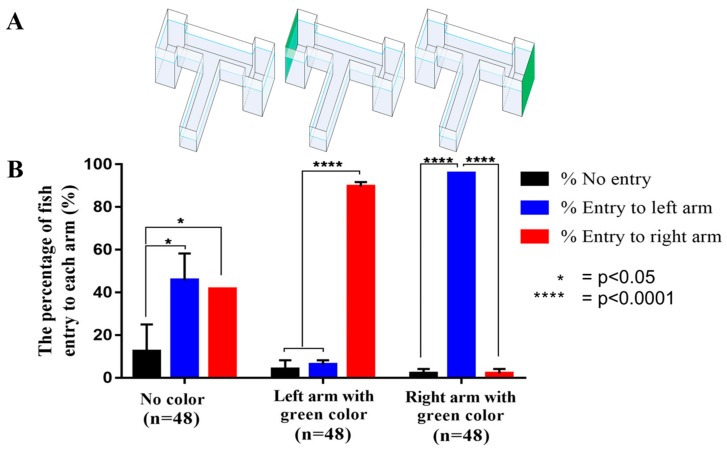
The assessment of individual fish with color cue option in the habituation phase. (**A**) Three T-maze designs. (**B**) The comparison of percentage fish entry to each arm in three different T-maze configurations. Based on the results, we decided to use the T-maze apparatus with a green cue at the right arm and electric shock at the left arm (punishment chamber). The data expressed as the means ± SEM. The data were analyzed by one-way ANOVA and followed with the Tukey–HSD test (* *p* < 0.05, **** *p* < 0.0001, *n* = 48).

Figure 2A shows the green cue stimulus, which is designed to increase the stimulus in one target arm. In the first experiment, Zones III and IV were maintained as transparent. Six-month-old adult wild type (WT) zebrafish were allowed to freely explore the inner space in habituation trials. After acclimation, the gate between Zones I and II was raised and fish were allowed to swim through the novel arm and into the chosen areas. Once fish approached the T-way intersection, there are three options, namely, entry into the left or right arm or no entry at all. To augment the tendency of the initial choice of the fish, we tested with two trials in parallel, which were a green cue that was given at either left or right arms. The time when fish stayed in Zones III and IV was examined and quantitatively compared between three experimental groups (Figure 2B). In Figure 2B, there was no significant difference regarding fish movement into left or right arms when the T maze was decorated with no color cue. However, when the green color cue was placed in either the left or right chamber, the majority (90–100%) of the tested fish displayed a significant increment of time spent in the opposite arm without color decoration (*p* < 0.0001, Figure 2B). Based on this interesting preliminary test, we decorated our T-maze apparatus with the green color cue at the right arm (Zone IV) for all the experiments conducted in this study. This specific setting could force the majority of the tested zebrafish to swim to the left punishment arm (Zone III) to increase the data reproducibility and shorten the training period.

### 2.3. Comparison of Learning Capacity and Memory Retention Between Male and Female Zebrafish

**Figure 3:**
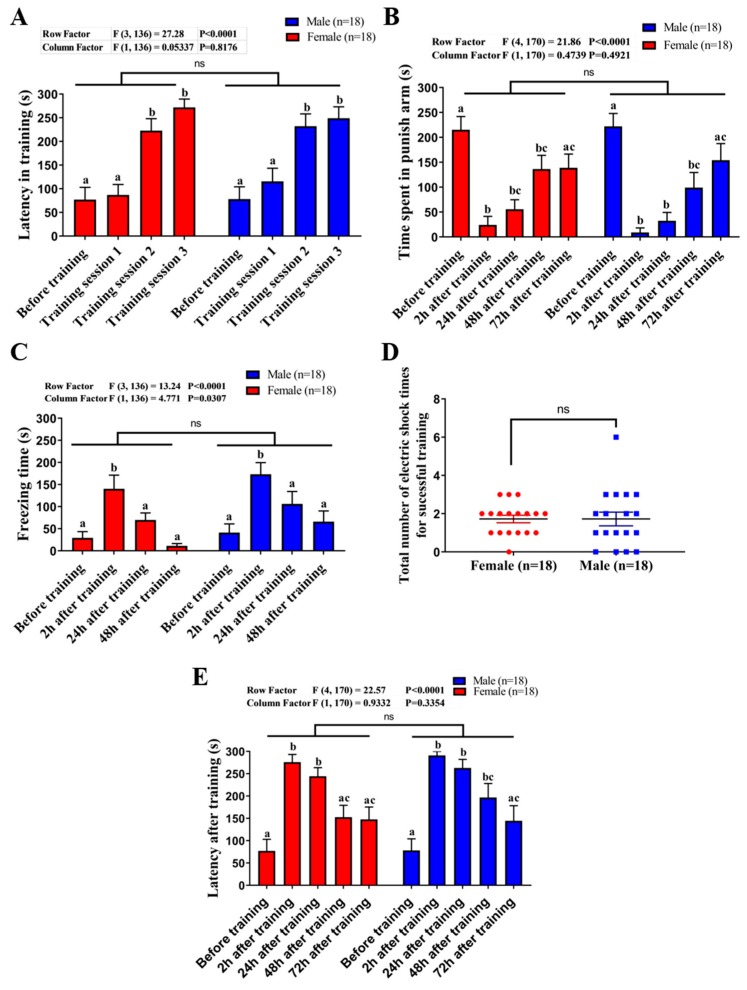
The assessment of the influence of gender on learning and memory in the T-maze test. (**A**) Comparison of the latency (s) for male (blue) and female (red) fish to swim into the punishment chamber for each training session. (**B**) Comparison of the time spent in punish arm (s) for male and female fish at different time points before and after training. (**C**) Comparison of the freezing time (s) for male and female fish at different time points before and after training. (**D**) Comparison of the total number of electric shock between male and female fish. (**E**) Comparison of the memory retention latency (s) for male and female fish at different time points after training. The data are expressed as means ± SEM. The data were analyzed by two-way ANOVA and followed with the Tukey–HSD test. Different letters (a, b, c) on the error bars represent significant differences (*p* < 0.05, *n* = 18).

We tested whether the learning and memory of the zebrafish are influenced by the difference between genders. We used sexually matured zebrafish at six to eight months old for testing. Five endpoints in terms of latency in the training phase, time spent in the punishment chamber, freezing time, the total number of electric shocks, and memory latency were determined to evaluate the learning capability and memory retention of zebrafish. Results showed that there were no significant differences between male and female zebrafish on latency in training (ANOVA F_1136_ = 0.05337, *p* = 0.8176, Figure 3A), time spent in the punishment chamber (ANOVA F_1170_ = 0.4739, *p* = 0.4921), freezing time (ANOVA F_1136_ = 4.771, *p* = 0.0307, Figure 3C), total number of electric shocks (Figure 3D), and latency after training (ANOVA F_1170_ = 0.9332, *p* = 0.3354, Figure 3E) tested by our modified T-maze setting. Therefore, the potential bias coming from animal genders can be ignored in the following experiments.

### 2.4. Comparison of Learning Capacity and Memory Retention Between Control and ZnCl_2_-Incubated Zebrafish

**Figure 4:**
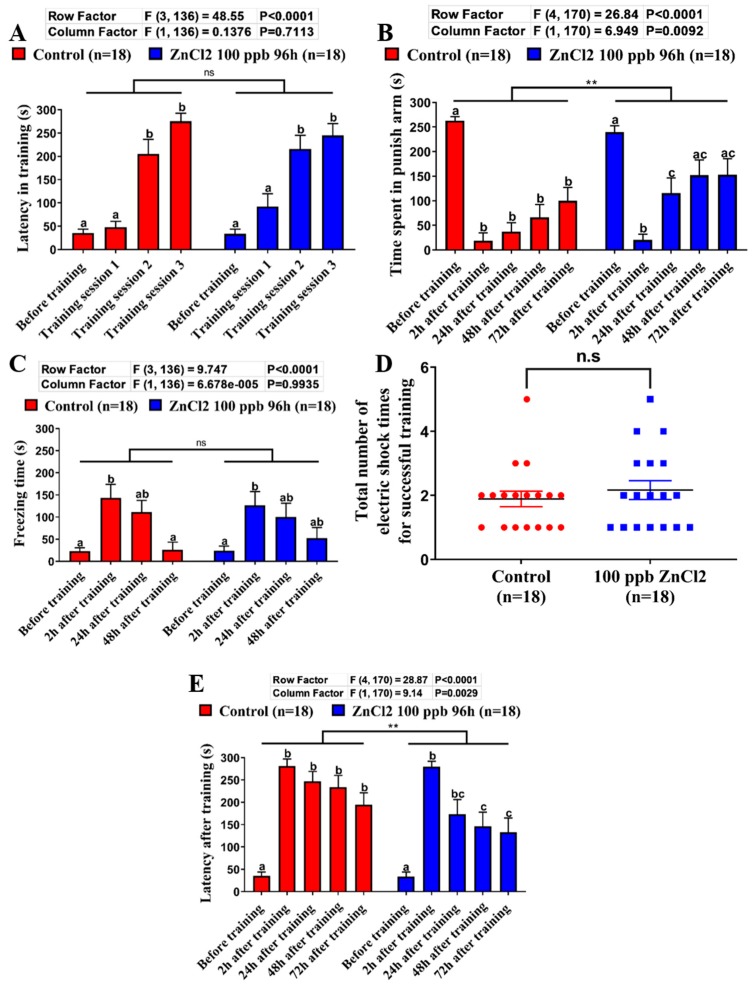

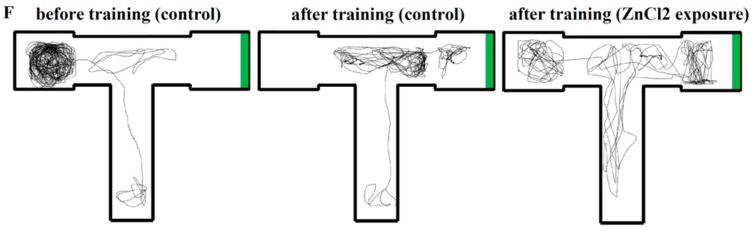
The assessment of toxicity of ZnCl_2_ exposure on learning and memory performances in the T-maze test. (**A**) The latency (s) of control and ZnCl_2_-incubated fish to swim into the punishment chamber for each training session. (**B**) The time spent in the punishment chamber (s) of control and ZnCl_2_-incubated fish at different time points (before and after training). (**C**) The freezing time (s) of control and ZnCl_2_-incubated fish at different time points before and after training. (**D**) The total number of electric shocks given for control and ZnCl_2_-incubated fish. (**E**) The memory retention latency (s) of control and ZnCl_2_-incubated fish at different time points after training. (**F**) The typical swim locomotion trajectories for control fish before (left panel) and after training (middle panel), and for ZnCl_2_ exposed fish after training (right panel). The data are expressed as means ± SEM. The data were analyzed by two-way ANOVA and followed with the Tukey–HSD test (** *p* < 0.01, *n* = 18). Different letters (a, b, c) on the error bars represent significant differences (*p* < 0.05).

In a previous study, we discovered that low concentrations of ZnCl_2_ could reduce the brain acetylcholine content and induce memory loss in zebrafish by using a passive avoidance shuttle box test [25]. However, in the passive avoidance test, there is a tendency that the zebrafish will swim in the dark area as an innate response (usually less than 5 s). Therefore, the passive avoidance test is not suitable to test learning performance in zebrafish. In this consideration, we aimed to use this modified T-maze to validate the learning and memory performances of ZnCl_2_-incubated fish. After four days of ZnCl_2_ exposure at 100 ppb, we used the modified T-maze to perform learning and memory tests. As shown in Figure 4A, the latency in training of the treatment group was significantly increased after training session 1 for both control and ZnCl_2_-incubated fish, but there were no significant differences in each training session in comparison with the control group (ANOVA F_1136_ = 0.1376, *p* = 0.7113). In addition, the total number of electric shocks of ZnCl_2_-incubated fish was also similar to the control group, which indicated that ZnCl_2_ exposure had no effect on the learning performance for escaping dangerous objects (Figure 4D). Furthermore, we found that there were no significant differences between the control and ZnCl_2_-incubated fish on the learning performance of stimulation avoidance. However, we discovered that the ZnCl_2_-incubated fish spent more time in the punish arm after training (Figure 4B,F, right panel; ANOVA F_1170_ = 6.949, *p* = 0.0092) as well as displayed a shorter latency for swimming to the punish arm on testing session (Figure 4E; F_1170_ = 9.14, *p* = 0.0029). The locomotion trajectories for zebrafish before and after ZnCl_2_ exposure were demonstrated in Figure 4F. These results demonstrate that ZnCl_2_ at a low concentration affects the memory capacity of zebrafish. Taken together, this modified T-maze process was able to detect ZnCl_2_-induced memory impairment in zebrafish.

### 2.5. Comparison of Learning Capacity and Memory Retention Between the Wild Type and Mutant Zebrafish

**Figure 5:**
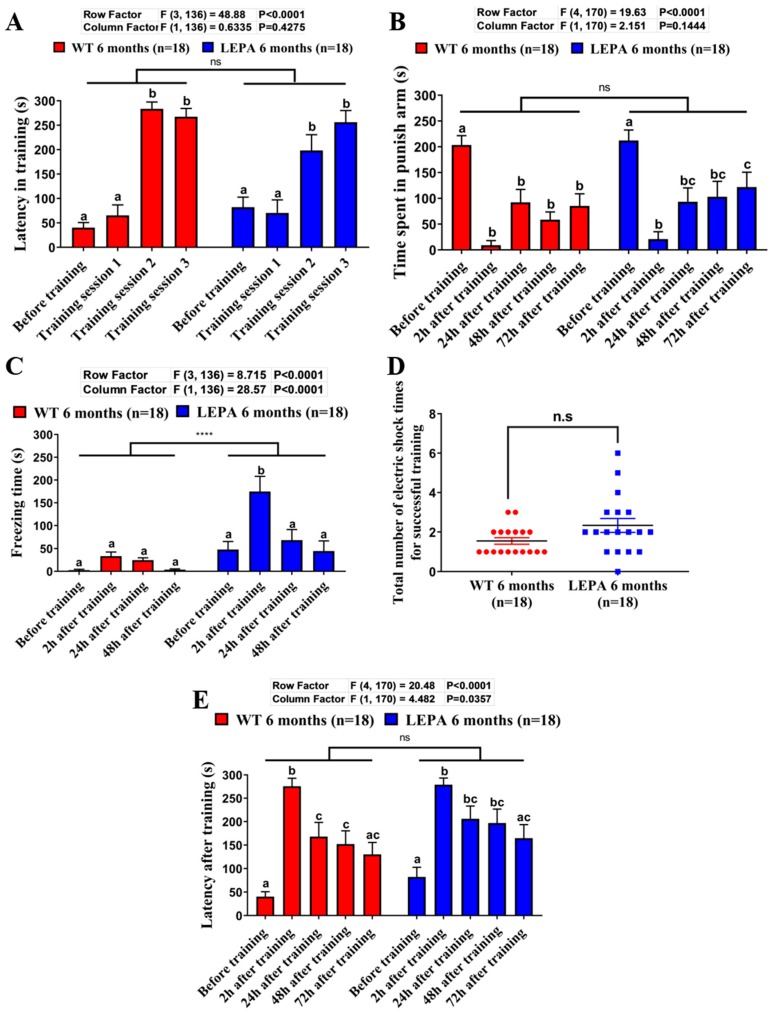
The comparison of learning and memory capabilities between WT and *lepa* KO fish. (**A**) Comparison of the latency (s) of control (red) and *lepa* KO (blue) fish to swim into the punishment chamber for each training session. (**B**) Comparison of the time spent in punish arm (s) for control and *lepa* KO fish at different time points before and after training. (**C**) Comparison of the freezing time (s) for control and *lepa* KO fish at different time points before and after training. (**D**) Comparison of the total number of electric shocks between control and *lepa* KO fish. (**E**) Comparison of the memory retention latency (s) for control and *lepa* KO fish at different time points after training. The data are expressed as mean ± SEM and bars with the same letter are not significantly different from each other. (**A**) the data were tested by one-way ANOVA with Tukey–HSD post hoc test; (**B**–**E**) the data were tested by two-way ANOVA with Tukey–HSD post hoc test (* *p* < 0.05, **** *p* < 0.0001, *n* = 18). Different letters (a, b, c) on the error bars represent significant differences (*p* < 0.05).

We also explored the utility of the modified T-maze protocol to evaluate the learning capacity and memory retention between wild type (WT) and mutant fish. Zebrafish mutants with a *lepa* gene deficiency have been described in our previous publication [26]. In that study, this mutant fish showed an obesity phenotype and multiple behavioral abnormalities. With the passive avoidance test, we found that memory retention between WT and *lepa* KO fish were different [26]. In this study, the learning and memory performances of *lepa* KO fish were re-evaluated by our current modified T-maze setting. From the result, we found that there were no significant differences in their latency in training (ANOVA F_1136_ = 0.6335, *p* = 0.4275, Figure 5A) and the total number of shocks needed in the training phase (Figure 5D). In addition, the number of shocks is correlated with memory acquisition in zebrafish. Further, a total of three training sessions were conducted and the results indicated that zebrafish needed multiple training sessions to increase their memory acquisition and at least two training sessions to reach a high latency in the training phase. The training phase is considered to be successful when all zebrafish lines are able to reach a significant difference in latency to avoid the punishment area before and after training time.

To evaluate the memory retention of zebrafish, the latency in the non-punishment area was measured. The results showed that zebrafish had the highest latency within 2 h after the training phase and the latency decreased over time after 24, 48, and 72 h. Furthermore, it was observed that the zebrafish could retain their memory for 48 h. After 72 h, there was no significant difference observed in comparison to the latency before training (Figure 5A). We also measured the time spent in the punishment arm (Figure 5B). Before training, all three groups showed the highest latency and the training phase was significantly reduced (F_4204_ = 31.97, *p* < 0.0001). Freezing time was considered as one of the parameters related to memory capability. In addition, freezing behavior has been associated with anxiety in zebrafish [27,28]. Figure 5C shows the average freezing time during the training phase in *lepa* KO fish. Longer freezing time in *lepa* KO zebrafish was observed to the WT zebrafish. (ANOVA F_1136_ = 28.57, *p* < 0.0001). We measured the latency in the testing phase and we found a slight difference between the *lepa* KO and WT fish after we tested with a two-way ANOVA test (with column factor F_1170_ = 4.482, *p* < 0.0035). However, after side-by-side comparison of the latency time in each different time points were conducted, we found that the short-term memory performance between *lepa* KO and control fish did not display any significant difference (Figure 5E).

## 3. Discussions

In this study, we present a modified T-maze protocol that shows several advantages, including shorter training and testing period (3-day protocol), small instrument size, and reproducibility. The specific design of our modified T-maze contained green color cue at the right arm and optimized electric shock at the left arm to facilitate zebrafish conditional training. In previous studies, a color cue was also introduced at one arm of the T-maze [29], three-side sleeves [30], or at the bottom of the maze [31]. We found that one green sheet placed at the non-punishment arm was enough to force zebrafish to swim to the left arm during the training phase with a 90–100% success rate (Figure 2B). Therefore, the arm with no color cue was chosen as the punishment arm to converse the zebrafish selection against its preferred choice by using mild electric shock.

Our modified T-maze was designed with a shorter novel arm compared to previous studies (summarized in Table S1), which allowed us to minimize the operational difficulties, thus increasing the experimental throughput when multiple T-mazes were operated simultaneously. Generally, a larger number of fish are required in group habituation to reduce the acute social isolation stress especially with a longer and more complex maze design. In our modified T-maze design, we achieved a shorter latency to the target arm to reduce the acclimation duration and the entire training period. Also, as reported by Aoki et al., electric shock avoidance and Y-mazes were also used for memory tests in zebrafish [31]. However, due to the lack of spatial cues in the initial training phase, up to 120 training trials are required.

Since the training phase of our modified T-maze is facilitated by mild electric shock punishment, one potential risk of our protocol is that zebrafish might be frozen due to the high level of anxiety that can be caused by electric shocks. This freezing behavior might lead to longer latency time during the testing phase and cause the overestimation problem for memory retention. To avoid this problem, the mild electric shock conditions were optimized as 1–2 V voltage and 0.3–0.5 mA electric currents. In addition, the freezing time during the training phase should be recorded to monitor the anxiety level from time to time. For example, compared to the WT fish, it was determined that the *lepa* KO fish displayed higher freezing times during the training phase (Figure 5C). This observation is consistent with our previous publication, which showed a high anxiety level observed when the *lepa* gene was compromised in zebrafish [26]. Taken together, for the first time, we have established a three-day T-maze protocol that can be used to evaluate the anxiety, learning, and memory capacity of adult zebrafish. In addition, there is a high potential for this modified T-maze to be applied with other emerging technologies, such as ones related to microfluidics, the science and technology of manipulating nanoliter volumes in microscale fluidic channels [32]. Since the microfluidic technology also offers a growing set of tools for manipulating small volumes of fluids to control chemical, biological, and physical processes that are relevant to sense, we certain that this technology can improve the modified T-maze by conducting higher controlled experiments. Moreover, its automation may also improve the current modified T-maze in terms of chemical manipulation processes [33,34].

## 4. Materials and Methods

### 4.1. Animals

Wild type (WT) AB strain and *lepa* KO fish were kept in the zebrafish core facility of Chung Yuan Christian University, Taiwan. Adult fish were maintained in a flow-through facility filled with filtered and dechlorinated tap water at approximately 28 °C. Light and dark cycles followed the standard environmental condition and stabilized by light tubes at the ceiling for 14 h (08:00–22:00). Fish were fed twice a day at 9:00 a.m. and 4:00 p.m., with flake food or freshly hatched brine shrimp. Before testing with T-maze, fish were transferred into individual tanks (160 mm × 90 mm × 70 mm) with 30% water changed daily. The Committee for Animal Experimentation of the Chung Yuan Christian University approved all of the experimental protocols and procedures involving zebrafish (Number: CYCU107030, issue date 19 Dec. 2018). All of the experiments were performed in accordance with the guidelines for laboratory animals.

### 4.2. ZnCl_2_ Exposure Incubation

Zinc chloride (ZnCl_2_) was purchased from Sigma-Aldrich Corp. (St. Louis, MO, USA). Thirty-six WT (AB) adult fish aged around six-month old were divided into two groups (with 18 fish each) and kept in 10-L tanks with oxygen supply by air pumping. The first group without treatment of ZnCl_2_ served as control group. The treatment group was waterborne exposed to 100 ppb ZnCl_2_ solution for 96 h according to our previous tested condition [25]. After ZnCl_2_ incubation, fish were washed three times with excess water and then transferred into individual tanks for memory testing by using a T-maze.

### 4.3. T-Maze Paradigm

Our T-maze was designed based on a study described by Braida et al. [24], with some modifications. The modified T-maze apparatus used in this study is made from 3 mm thin transparent Plexiglas and includes two deep-water arms and one straight long arm in which the inner space was divided into a T-way intersection (Figure 1A). The straight tunnel (300 mm × 80 mm × 60 mm) is divided into the starting chamber (Zone I) and the novel arm (Zone II) by a white-colored gate. The novel arm branched into two short arms (80 mm × 65 mm × 60 mm) leads to the deeper left/right chambers (100 mm × 100 mm × 100 mm) at a three-way junction. The apparatus (35 mm deep water) included Zones I, II (start chamber and novel arm) and Zones III and IV (deep water chambers) at two sides of each T-way section, which was filled with 85 mm deep water (Figure 1B). An electric stimulus (1–2 V, 0.3–0.5 mA) is given in Zone III (left arm). In addition, based on conditioned place preference behavior reported in adult zebrafish [30], one wall of Zone IV (right arm) was designed with a green color cue [35] to force zebrafish to swim to Zone III. A CCD camera for video recording (OPTO, CCD, China) was fixed at 60 cm height above the T-maze and connected to a personal computer workstation with NCH Debut Video software. A LED platform (450 mm × 450 mm × 5 mm) was placed at the bottom of the T-maze to provide uniform background light intensity and enhance image contrast. Then, the X and Y coordinates of fish locomotion were tracked using idTracker software [36].

### 4.4. Protocol

The zebrafish underwent a habituation trial to minimize apparatus novelty stress [24]. In the habituation group trials, six fish were placed in a starting chamber for one minute before the gate was opened and fish were allowed to freely explore the entire T-maze for 30 min. Before individual habituation trials, fish were placed into individual 600 mL plastic tanks to acclimate them to isolated conditions for two hours. Then, each fish was placed in a starting chamber with its gate closed. After 30 s, the gate was opened and the fish was allowed to freely explore the T-maze. After the individual habituation trial, the fish were returned to individual plastic tanks for 24 h prior to training. The training phase for each fish consisted of three sessions with a maximum of three electric shocks given per session. In every session, each fish was placed in the starting chamber (Zone I) for 30 s and then allowed to freely swim in the maze. The fish that entered the left arm immediately received a mild electric shock (1–2 V and 0.3–0.5 mA). If it did not escape from the left arm after 20 s, the fish would be retrained. Each training session ended without any fish entering the left arm within 5 min. Next, the fish was removed from the apparatus and returned to its housing tank until the testing phase which has the same protocol as the training trials but without the electric shock unit. The latency to enter the left arm and the time spent in the punishment arm during each trial were recorded. Also, the expected increment in the testing trials was used as an index to evaluate learning capability and memory retention of fish.

### 4.5. Statistical Analysis

The data were analyzed using one-way ANOVA or two-way ANOVA depending on experiment design and followed by the Tukey–HSD test. The statistical analysis and data visualization was carried out with GraphPad Prism 7.00 for Windows. The data shown are presented with mean ± SEM with *p* < 0.05 regarded as statistically significant.

## Supplementary Materials

Supplementary Materials can be found at https://www.mdpi.com/1422-0067/21/4/1464/s1.
