# Supplementary material for: Development of a Modified Three-Day T-maze Protocol for Evaluating Learning and Memory Capacity of Adult Zebrafish
Source: Int J Mol Sci. 2020 Feb 21;21(4):1464. doi: 10.3390/ijms21041464 (PMC7073029; doi:10.3390/ijms21041464)
Supplement: Supplementary file 1 [file ijms-21-01464-s001.pdf]

Table S1. Comparison of this study with previous other type of maze on evaluating learning and memory performance in zebrafish

Design	Age-zebrafish	Traveled length	Stimulus	Duration	Citation
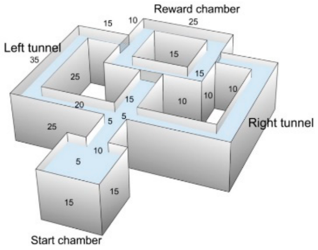	Adult	90 cm	Conspecific	16 days	[1]
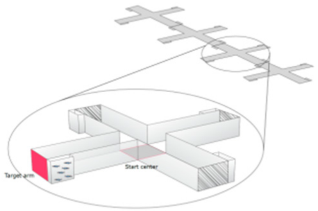	Adult	11.25 cm	Color cue and conspecific	8 days	[2]
- The stem of the maze: 50 x 10 x 10 cm,<br>- Area at the foot of the stem to form a star box: 10 x 10 x 10 cm<br>- Each arm of the maze: 20 x 10 x 10 cm	Adult	40cm	Color cue and food	Unknown	[3]

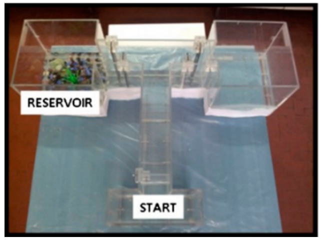	Adult	50cm	Preference cue and food	6 days	[4]
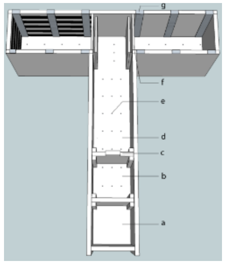	Adult	40cm	Electric shock	4 days	[5]
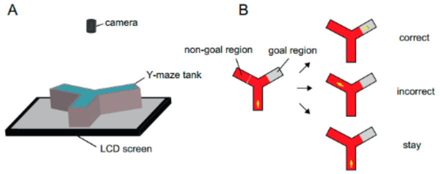	Adult	20 cm	Color cue (bottom) and electric shock	120 trials/2 day	[6]
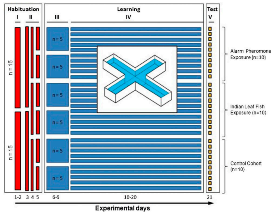	Adult	50 cm	Color cue and food	21 days	[7]

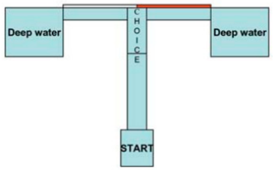	Young-middle	14 cm	Color cue and food	Unknown	[8]
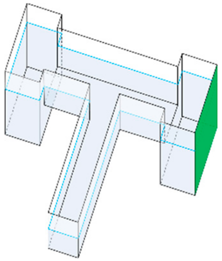	Adult	22 cm	Color cue and electric shock	9 trials/2 days	This study

1. Naderi, M.; Jamwal, A.; Ferrari, M.C.; Niyogi, S.; Chivers, D.P. Dopamine receptors participate in acquisition and consolidation of latent learning of spatial information in zebrafish (danio rerio). *Progress in Neuro-Psychopharmacology and Biological Psychiatry* **2016**, *67*, 21-30.
2. Naderi, M.; Salahinejad, A.; Ferrari, M.C.; Niyogi, S.; Chivers, D.P. Dopaminergic dysregulation and impaired associative learning behavior in zebrafish during chronic dietary exposure to selenium. *Environmental Pollution* **2018**, *237*, 174-185.
3. Colwill, R.M.; Raymond, M.P.; Ferreira, L.; Escudero, H. Visual discrimination learning in zebrafish (danio rerio). *Behavioural Processes* **2005**, *70*, 19-31.
4. Braida, D.; Ponzoni, L.; Martucci, R.; Sparatore, F.; Gotti, C.; Sala, M. Role of neuronal nicotinic acetylcholine receptors (nachrs) on learning and memory in zebrafish. *Psychopharmacology* **2014**, *231*, 1975-1985.
5. Saili, K.S.; Corvi, M.M.; Weber, D.N.; Patel, A.U.; Das, S.R.; Przybyla, J.; Anderson, K.A.; Tanguay, R.L. Neurodevelopmental low-dose bisphenol a exposure leads to early life-stage hyperactivity and learning deficits in adult zebrafish. *Toxicology* **2012**, *291*, 83-92.
6. Aoki, R.; Tsuboi, T.; Okamoto, H. Y-maze avoidance: An automated and rapid associative learning paradigm in zebrafish. *Neuroscience research* **2015**, *91*, 69-72.
7. Gaikwad, S.; Stewart, A.; Hart, P.; Wong, K.; Piet, V.; Cachat, J.; Kalueff, A.V. Acute stress disrupts performance of zebrafish in the cued and spatial memory tests: The utility of fish models to study stress–memory interplay. *Behavioural Processes* **2011**, *87*, 224-230.
8. Yu, L.; Tucci, V.; Kishi, S.; Zhdanova, I.V. Cognitive aging in zebrafish. *PloS one* **2006**, *1*, e14.
